# Supplementary material for: The Australian Eye and Ear Health Survey (AEEHS): Study protocol for a population-based cross-sectional study
Source: PLoS One. 2024 May 31;19(5):e0301846. doi: 10.1371/journal.pone.0301846 (PMC11142534; doi:10.1371/journal.pone.0301846)
Supplement: Attachment — Submitted filename: The Australia Eye Survey.docx [file pone.0301846.s002.docx]

The Australia Eye Survey

Comments

**General**

The survey protocol is interesting to read. One important argument is, that the findings from the survey of 5,000 individuals will represent the whole of Australia, which has a population of nearly 26 million. Although the estimation of sample size was done scientifically along the scientific way of sampling, the readers will be skeptical about the representativeness and generalizability of the findings. Maybe the authors can share their justification and thoughts on this. This will help many researchers.

**Specific**

There is a lack of clarity in the sample size estimation.

The author can share the parameters used in the estimation of sample size from the previous survey. This will make clarity to the readers.

Again the author mentioned that….. “to detect a 2.87% difference in visual impairment prevalence….” Was the sample size estimation done based on the difference in the prevalence of visual impairment between the previous and current surveys? The author made an assumption that keeping 2.87% of the difference in the prevalence, what should be a sample size with 99% power? Needs clarification.

The author can give reasons why indigenous were being oversampled (3.2%). When one sub-population of the whole being sampled, the sample may not be representative and findings may not be generalizable. How did the author decide 3250 non-indigenous and 1750 individuals from Indigenous?

Regarding site selection, the author can explain more about SA 1 and SA 2. How many SA1 and SA2 are in the whole of Australia? Maybe the author can incorporate a flow chart of Multi-stage cluster random sampling. The author can mention clearly what cluster size and numbers to be selected for each non-indigenous and indigenous Australian.

The cluster size was 100 for non-indigenous and 50 for indigenous, to be selected from each of 30, then the numbers did not reach the estimated sample number.

First stratification is done based on state and territory, how many states and territories are in Australia? How did the number of sites being decided? (E.g., New South Wales, 6 from low and 4 from high in Table 1). This can be shared.

The author can share details of the survey team and their responsibilities. It is worth highlighting the training undergone by the team. Whether the authors plan for any estimation of agreement among teams in terms of examination skills.

**Data management and storage**

Maybe the author can highlight how data entry, checking, and cleaning will be done. I think data collection is planned in physical paper format, not e-format. This part is worth mentioning.

The author can also share the expected key outcomes of the survey which you think are the most important.
