# Supplementary material for: The Australian Eye and Ear Health Survey (AEEHS): Study protocol for a population-based cross-sectional study
Source: PLoS One. 2024 May 31;19(5):e0301846. doi: 10.1371/journal.pone.0301846 (PMC11142534; doi:10.1371/journal.pone.0301846)
Supplement: Attachment — Submitted filename: AUS_Study.docx [file pone.0301846.s004.docx]

Comments

Thank you to all the authors for the revision that was done nicely, especially the sample size estimation in each survey site.

I have a few minor comments now.

On page 15, lines 1 and 3, “the study team comprises” was used two times. The author can paraphrase it.

**NOTE:**

(The following is the argument among us, within scientific communities, not to be included in the manuscript)

Regarding the issue of sample size whether it is representative of a country or not, my suggestion or personal experience is even if we used all robust scientific methods for the sample size estimation**, for advocacy purposes**, the decision or policymakers usually considered a study with a bigger sample size. For example, hypothetically a separate team conducts a similar or the same study with the same objectives with a bigger sample size (N=15000) using the same sampling technique, the policymaker prefers the study with the bigger sample size to be used for the planning and policy purposes. Additionally, our goal of population-based study is for advocacy purposes.

Even “face validity on the representativeness” also, the study with a bigger sample seems more valuable.

Therefore, I feel sometimes that face validity matters.
