# Supplementary material for: The Australian Eye and Ear Health Survey (AEEHS): Study protocol for a population-based cross-sectional study
Source: PLoS One. 2024 May 31;19(5):e0301846. doi: 10.1371/journal.pone.0301846 (PMC11142534; doi:10.1371/journal.pone.0301846)
Supplement: Attachment — Submitted filename: AEEHS Protocol Paper Reviewer Comments Response.docx [file pone.0301846.s003.docx]

15 Feb 2024

To the Editor

Dear Dr Pruller,

MS: PONE-D-23-19954

Thank you for the opportunity to revise our manuscript above and respond to the reviewer’s comments below. Changes to the manuscript are highlighted with tracked changes. We hope it is now acceptable for publication.

Please do contact me if there is anything else we can do to improve the manuscript.

Sincerely,

Dr Richard Kha on behalf of all authors

**Reviewer 1 Comments**

***Comment 1.*** *The survey protocol is interesting to read. One important argument is that the findings from the survey of 5,000 individuals will represent the whole of Australia, which has a population of nearly 26 million. Although the estimation of sample size was done scientifically along the scientific way of sampling, the readers will be skeptical about the representativeness and generalizability of the findings. Maybe the authors can share their justification and thoughts on this. This will help many researchers.*

**Response 1.** We acknowledge the reviewers’ comment but would like to respectfully disagree. Approximately 5000 individuals, carefully selected to be representative, is the usual sample size for national population studies with similar aims. For example, the highly regarded National Health and Nutrition Examination Survey (NHANES), run by the Centers for Disease Control, USA, has been conducting regular surveys of the US population since 1960s. It recruits 5000 participants from 15 counties, and the data is accepted to be representative of the entire US population of 330 million. (https://www.cdc.gov/nchs/nhanes/about_nhanes.htm)

The 1st National Eye Health Survey (NEHS1), which is the predecessor to our current survey, also surveyed almost 5000 participants from 30 sites, using a similar methodology, and the results are accepted to be generalizable to the wider Australian population. We are confident the results will be representative and generalizable to the wider Australian population.^1,2^

^1^ Foreman J, Xie J, Keel S, van Wijngaarden P, Sandhu SS, Ang GS, Fan Gaskin J, Crowston J, Bourne R, Taylor HR, Dirani M. The Prevalence and Causes of Vision Loss in Indigenous and Non-Indigenous Australians: The National Eye Health Survey. Ophthalmology. 2017 Dec;124(12):1743-1752. doi: 10.1016/j.ophtha.2017.06.001. Epub 2017 Jul 6. PMID: 28689897.

^2^ Keel S, Xie J, Foreman J, van Wijngaarden P, Taylor HR, Dirani M. Prevalence of Age-Related Macular Degeneration in Australia: The Australian National Eye Health Survey. JAMA Ophthalmol. 2017 Nov 1;135(11):1242-1249. doi: 10.1001/jamaophthalmol.2017.4182. PMID: 29049463; PMCID: PMC5710385.

***Comment 2.*** *There is a lack of clarity in the sample size estimation. The author can share the parameters used in the estimation of sample size from the previous survey. This will make clarity to the readers. Again the author mentioned that….. “to detect a 2.87% difference in visual impairment prevalence….” Was the sample size estimation done based on the difference in the prevalence of visual impairment between the previous and current surveys? The author made an assumption that keeping 2.87% of the difference in the prevalence, what should be a sample size with 99% power? Needs clarification.*

**Response 2.** We apologise for the lack of detail and unclear phrasing, we now provide more detail which will hopefully address the reviewer’s concerns.

**Page 6, Lines 11-16.** “Assuming a statistical significance level of 0.05 and sampling of 1,750 Indigenous and 3250 non-Indigenous Australians as in the NEHS1, we will have a power >99% to detect the same difference in Vision Impairment prevalence that has been previously reported (gap of 4.7%, i.e. 11.2% vs 6.5% respectively), in NEHS1 findings [13]. Further, we will have 90% power to measure a narrowing of the gap in Vision Impairment prevalence between the two groups of 2.87% or wider.”

***Comment 3.*** *The author can give reasons why indigenous were being oversampled (3.2%). When one sub-population of the whole being sampled, the sample may not be representative and findings may not be generalizable. How did the author decide 3250 non-indigenous and 1750 individuals from Indigenous?*

**Response 3.** We thank the reviewer for this important comment and the opportunity to provide further details. We now describe this in further detail. Our power calculations below indicated a minimum number of 1500 Indigenous participants needed to be sampled, in order to detect a 2% margin of error in the estimated vision impairment prevalence of 17.2%. This was increased to 1750 to account for ungradable images. Although technically an oversampling, this was driven by the power calculations, and needed to obtain reliable estimates with narrow confidence intervals. We now realise that describing this as ‘oversampling’ confuses the issue and have now deleted this sentence.

**Page 5, Line 19 to Page 6, Line 9.** “This study is a follow-up study of the NEHS1, thus the sample sizes were determined from the NEHS1 to ensure comparability.

The sample size calculation assumed a similar prevalence of vision impairment in non-Indigenous Australians to the pooled prevalence reported in previous studies, the Melbourne Vision Impairment Project [19] and the Blue Mountains Eye Study [20] of 5.2% and a prevalence of 17.2% in Indigenous Australians, as reported in the National Indigenous Eye Health Study [21].

Assuming a margin of error of 1.1% for non-Indigenous Australians and 2% for Indigenous Australians, a design effect of 1.5 that adjusted for interclass correlations and a 20% non-response rate, the required sample size for the non-Indigenous and Indigenous populations was approximately 3000 and 1500, respectively. Based on this sample size, and an expected cluster of 100 non-Indigenous individuals and 50 Indigenous individuals in each site, 30 recruitment sites were required [22]. In order to correct for an estimated 8% non-Indigenous, and 16% Indigenous rate of ungradable images (due to cataract, poor compliance, corneal opacity etc [23]), sample size for non-Indigenous and Indigenous populations was increased to 3250 and 1750 participants respectively.”

***Comment 4.*** *Regarding site selection, the author can explain more about SA 1 and SA 2. How many SA1 and SA2 are in the whole of Australia? Maybe the author can incorporate a flow chart of multi-stage cluster random sampling. The author can mention clearly what cluster size and numbers to be selected for each non-indigenous and indigenous Australian. The cluster size was 100 for non-indigenous and 50 for indigenous, to be selected from each of 30, then the numbers did not reach the estimated sample number. First stratification is done based on state and territory, how many states and territories are in Australia? How did the number of sites being decided? (E.g., New South Wales, 6 from low and 4 from high in Table 1). This can be shared.*

**Response 4.** Thank you. Please see response above for explanation of how cluster sizes of 100 and 50 were obtained, and how this was increased to reach the total sample of 5000.

The other comments are addressed below, in the revised manuscript.

**Page 7, Line 1 to Page 8, Line 7.** “Multi-stage, random-cluster sampling was used to select 30 geographic areas to provide a representative sample. The first stage of sampling involved selecting sites at the Australian Statistical Geography Standard (ASGS) Statistical Area Level 2 (SA2) areas [14]. SA2 geographic areas refer to population clusters of approximately 10,000 residents. In turn, SA2 clusters are comprised of smaller SA1 geographic units, each of which contains approximately 200–800 persons. There are 2097 SA2s and 57,523 SA1s in Australia [14].

A second level of stratification was employed based on Indigenous status. Each state and territory had the SA2 sites allocated to a high Indigenous proportion group and a low Indigenous proportion group. This was done to ensure the target of at least 1750 Indigenous and 3250 non-Indigenous persons will be achieved, due to the lower overall prevalance of Indigenous individuals. An 80th percentile cut-off (per state) was used to determine candidacy for the low and high Indigenous status group. This resulted in 12 high Indigenous proportion SA2 sites, and 18 low Indigenous proportion SA2 sites, approximating the ratio of 1750 to 3250 participants.

A third level of stratification was based on remoteness area as defined by the ASGS, in order to achieve a similar distribution of the Australian population according to whether they lived in cities or more rural locations. This determined that the SA2 sites should be distributed as 19 Major City, 7 Inner Regional, 3 Outer Regional and 1 Remote/Very Remote sites. At this stage, the number of sites per state and territory was allocated to be approximately proportional to the Australian population living in that state/territory. This was done to ensure representativeness and improve generalisability to the overall national population. Once these strata were achieved, the required number of SA2 sites by state/territory were randomly selected from randomly ordered lists of the relevant SA2s.

Within each selected SA2, a constituent SA1 or cluster of SA1s was nominated. Choice of SA1 was constrained by several feasibility criteria, primary among them was the availability of an examination site during the study period that the team would be in the state/territory. Accessibility was the other main criteria, as the study equipment had to be transported by van, and the examination site had to be accessible via sealed roads. If no suitable SA1 could be secured in that SA2, the next back up SA2 for that state/territory was considered from the randomly ordered list described above.”

***Comment 5.*** *The author can share details of the survey team and their responsibilities. It is worth highlighting the training undergone by the team. Whether the authors plan for any estimation of agreement among teams in terms of examination skills.*

**Response 5.** Thank you for the comment. We have added a section in the methods to detail the survey team and their responsibilities.

**Page 15, Lines 1-17.** “The study team comprises a Project Manager, with overall supervision and responsibility for study conduct, execution, hiring, training of staff and collection of high-quality data. The survey team comprises door knockers with responsibility for door knocking. This usually numbers between 2-6, depending on availability, and are hired locally and provided with 1 day training by the Project Manager or delegate. Three full time research officers with prior ophthalmic or hearing experience, supplemented by PhD students, conduct most of the examinations, under supervision of the Project Manager who also participates to ensure high quality data collection. The current research officers are optometry, orthoptic (ophthalmic nursing) and hearing science graduates. All examination staff undergo 2 weeks of training in good research conduct, familiarity with the Study Protocols, training in visual acuity and subjective refraction measurement, use of the imaging equipment, storage and backup of data, and emphasis on high quality data collection and maintaining participant confidentiality. Data collection is reviewed monthly by the study Biostatistician to identify missing data or inaccuracies, and remedial training and feedback is provided to the relevant staff member if this is identified. This regular oversight ensures consistency among data collection and examination skills of research officers. In cases of disagreement, arbitration is provided by the Study Principal Investigators with clinical experience (PM, GL).”

***Comment 6.*** *Maybe the author can highlight how data entry, checking, and cleaning will be done. I think data collection is planned in physical paper format, not e-format. This part is worth mentioning.*

**Response 6.** Thank you for the comment. We have expanded the data storage section in the manuscript.

**Page 15, Line 24 to Page 16, Line 7.** “Data entry, checking and cleaning are conducted in 2 stages. Initially at the end of each examination day, the Project Manager reviews data collected to ensure no missing data or anomalous values are recorded. If any issues are identified, these can be rectified on the same day. Every month, or more frequently if necessary, the Biostatistician reviews the data collected to check for consistency and any issues detected are immediately raised and corrected.

Data collection for most stations is done via entry into an electronic tablet or computer, directly into the database. Real time oversight can thus be provided by the Project Manager and Biostatistician.”

***Comment 7.*** *The author can also share the expected key outcomes of the survey which you think are the most important.*

**Response 7.** Thank you for the comment. We have now added the expected key outcomes of the survey in the Introduction section.

**Page 4, Lines 13-22.** “The key objectives of this study are:

- To establish the current prevalence, causes, risk factors and impacts of visual and hearing impairment in Australian adults.
- To identify the modifiable and non-modifiable risk factors for vision and hearing loss in adults.
- To analyse trends in eye health since the 2016 NEHS1 and report key metrics for universal eye health coverage e.g. effective cataract surgery coverage, effective refractive error coverage.
- Compare the prevalence of visual and hearing loss between Indigenous and non-Indigenous Australians, and between each urbanisation level.”

**Reviewer 2 Comments**

***Comment 1.*** *In the introduction, 2nd para, pls ensure that the economic cost for VI and HI are correct as HI is >3 times that of VI and also hearing aids are way more expensive than VI treatment esp. cataract sx and UCRE.*

**Response 1.** Thank you for the comment. This has been changed to a more recent reference which has an updated estimate of the cost of hearing impairment in Australia.

Page 3, Lines 12-13. “In Australia, the economic impact of vision and hearing loss is $27.6 and $33.3 billion per year, respectively [3, 5-7].”

***Comment 2.*** *The study will track changes in eye health with NEHS1. Would be good to clarify if the protocols are aligned between 2 studies for comparison.*

**Response 2.** Thank you for the comment. The sample size of 3250 non-Indigenous and 1750 Indigenous Australians remains the same as NEHS1. However, some of the protocols are different because this survey aims to improve upon the first survey by using new and more objective, comprehensive testing methods such as OCT, ilated fundus photography and Humphrey visual field testing, which was not performed in the NEHS1. We will also obtain additional data by using the autorefractor, IOLMaster and more comprehensive questionnaires to capture critical data. Protocols for NEHS1 for similar outcomes such as visual acuity were followed and expanded upon e.g. subjective refraction was also performed in this survey, to ensure comparability while also obtaining extra data.

***Comment 3.*** *For SS calculation, was prevalence of HI also taken into consideration? If not, why? Pls clarify.*

**Response 3.** Thank you for the comment. The prevalence of hearing impairment was not taken into account in the original sample size calculation as the hearing component of the study was added after the original eye health survey was funded. This occurred because the investigators were able to secure additional funding through other sources to conduct the hearing components of the study. The investigators have previously conducted combined vision and hearing studies, and this was a natural extension to the current eye survey.^3,4^

^3^ Gopinath B, McMahon CM, Burlutsky G, Mitchell P. Hearing and vision impairment and the 5-year incidence of falls in older adults. Age Ageing. 2016 May;45(3):409-14. doi: 10.1093/ageing/afw022. Epub 2016 Mar 5. PMID: 26946051.

^4^ Schneider J, Gopinath B, McMahon C, Teber E, Leeder SR, Wang JJ, Mitchell P. Prevalence and 5-year incidence of dual sensory impairment in an older Australian population. Ann Epidemiol. 2012 Apr;22(4):295-301. doi: 10.1016/j.annepidem.2012.02.004. Epub 2012 Mar 3. PMID: 22382082..

***Comment 4.*** *Under site selection (page 6), need reference for eye diseases in Aboriginal and Torres.*

**Response 4.** Thank you for the comment. References have been added for the statement regarding Aboriginal and Torres Strait Islander people in the aforementioned section.

Page 6, Lines 10-13. Aboriginal and Torres Strait Islander participants have a younger inclusion age criteria because major eye and hearing conditions such as diabetic retinopathy (DR) and glue ear have an earlier onset and faster rate of progression in these communities [18, 19].

***Comment 5.*** *Were people with cognitive impairment/dementia excluded as they may be unable to provide consent?*

**Response 5.** Yes, people with cognitive impairment and dementia may not have the capacity to provide informed consent and hence were excluded from the study.

***Comment 6.*** *How about blind/deaf? Were they excluded too?*

**Response 6.** Blind/deaf people are not excluded from the criteria as long as they are able to provide informed consent. Blind/deaf people are welcome to bring a support person along with them to help them sign the form. Additionally, the research team can read out the information to the participant and they can sign the consent form.

***Comment 7.*** *Recruitment: Each home will be visited twice. Any particular reason for visiting 2 times only? Usual practice is to approach at least 4 times before they are deemed uncontactable to ensure maximum participation and good response rate.*

**Response 7.** Thank you for the comment. This was to ensure compatibility with the recruitment methods of the first National Eye Health Survey (NEHS1) 7 years earlier, which visited each home twice.^5^ This was also most feasible from a practical and economic point of view, as our practical experience previously showed third and subsequent visits had diminishing returns.

^5^ Foreman J, Keel S, van Wijngaarden P, Taylor HR, Dirani M. Recruitment and Testing Protocol in the National Eye Health Survey: A Population-Based Eye Study in Australia. Ophthalmic Epidemiol. 2017 Dec;24(6):353-363. doi: 10.1080/09286586.2017.1296166. Epub 2017 Mar 27. PMID: 28346038.

***Comment 8.*** *A voluntary take home interview will be provided....why voluntary? Wouldn't it be better to administer on-site or make it mandate to get critical/rich data? Any previous experience of voluntary take-home uptake?*

**Response 8.** Thank you for the comment. The take-home questionnaire consists of several validated questionnaires, including a comprehensive dietary questionnaire. Although we would have preferred these questions to be administered onsite, this would have extended the testing duration to 2 hours or longer, which would affect recruitment and participation. Since the critical data is administered in the onsite questionnaires, we decided to add the remaining questionnaires as a voluntary take-home task to capture additional data. We have utilised voluntary take home questionnaires previously in other studies with good response rates (>65%).^6^

^6^ Nguyen H, Di Tanna GL, Do V, Mitchell P, Liew G, Keay L. 15-year incidence of driving cessation and associated risk factors: The Blue Mountains Eye Study. Maturitas. 2023 Nov;177:107796. doi: 10.1016/j.maturitas.2023.107796. Epub 2023 Jul 13. PMID: 37454471.

***Comment 9.*** *Why was DVA checked at 2 and not 4 m?*

**Response 9.** DVA was tested at 4 m, but in a 2-3m long room using electronic ETDRS charts that are calibrated to take into account the testing distance. This had to be done due to difficulty in securing short-term venues in Australia with enough room length to conduct vision testing at 4m. We have now updated the methods to explain this.

Page 10, Line 24 to Page 11, Line 1. “Distance visual acuity (DVA) – Presenting DVA will be measured at 4m using calibrated, electronic ETDRS LogMAR charts in well-lit room conditions, using habitual correction with spectacles or contact lenses, if worn.”

***Comment 10.*** *Gonioscopy is important for glaucoma diagnosis, but not included, why?*

**Response 10.** Thank you for the comment. We agree it would be ideal to include gonioscopy on all participants but this was not performed in the NEHS1, so to maintain consistency and alignment of protocols, we did not include it either as mandatory for all participants. We do perform the oblique pentorch test on all participants, and if occludable or narrow angles are identified, then anterior segment OCT and gonioscopy is performed in this subset of participants. This has been updated in the methods section.

Page 12, Lines 3-4. “If narrow angles are identified, then anterior segment OCT and gonioscopy will be performed on the participant.”.

***Comment 11.*** *I don't think this study will be the first study using OCTA on nationally representative sample. Pls cross check*

**Response 11.** Thank you for the comment. The statement has now been removed from the manuscript as another protocol paper was recently released which included OCT-A in a nationally representative sample.

***Comment 12.*** *Fundus photo done undilated or dilated, pls clarify.*

**Response 12.** The fundus photo is done dilated unless there are contraindications to dilation or the participant refuses dilation. This has been updated in the methods section.

Page 13, Line 13. “Mydriatic fundus photography – Ultra-widefield colour (200^°^ field of view)…”

***Comment 13.*** *Pls be consistent with the use of acronyms e.g. AMD/ DR etc. in the manuscript.*

**Response 13.** Thank you for the comment. The manuscript has now been edited accordingly to maintain consistency with acronyms.

***Comment 14.*** *What cut-offs will be used to define HI?*

**Response 14.** Thank you for the comment. The methods section has been updated to include the cut-offs used to define HI.

Page 14, Lines 15-17. “Hearing loss will be determined as the pure-tone average of audiometric hearing thresholds at 500, 1000, 2000 and 4000Hz, and any hearing loss will be defined hearing thresholds >20dB hearing level.”

***Comment 15.*** *Suggest including anthropometric measures, BP/HR before ocular tests to know any contraindication before dilation.*

**Response 15.** Thank you for the comment. We evaluate the iris anterior chamber angle grade with pentorch testing, and anterior segment OCT (in narrow angles) before pupillary dilation to minimise the risk of closed-angle glaucoma. Tropicamide is considered to be safe, with the literature showing that there is no statistically significant increase in blood pressure or heart rate after topical tropicamide.^7^

^7^ Vuori ML, Kaila T, Iisalo E, Saari KM. Systemic absorption and anticholinergic activity of topically applied tropicamide. J Ocul Pharmacol. 1994 Summer;10(2):431-7. doi: 10.1089/jop.1994.10.431. PMID: 8083562.

Hence, we chose to do anthropometric measurements after dilation drops to minimise examination time because it allows the pupils to dilate during station 2. This is also in line with previous studies we have conducted such as the Blue Mountains Eye Study.^8^

^8^ Wang JJ, Foran S, Mitchell P. Age-specific prevalence and causes of bilateral and unilateral visual impairment in older Australians: the Blue Mountains Eye Study. Clin Exp Ophthalmol. 2000 Aug;28(4):268-73. doi: 10.1046/j.1442-9071.2000.00315.x. PMID: 11021555.
